# Super-resolution microscopy reveals that Na^+^/K^+^-ATPase signaling protects against glucose-induced apoptosis by deactivating Bad

**DOI:** 10.1038/s41419-021-04025-8

**Published:** 2021-07-27

**Authors:** Kristoffer Bernhem, Jacopo M. Fontana, Daniel Svensson, Liang Zhang, Linnéa M. Nilsson, Lena Scott, Hans Blom, Hjalmar Brismar, Anita Aperia

**Affiliations:** 1 Science for Life Laboratory, Department of Applied Physics, Royal Institute of Technology, Solna, Sweden; 2 Science for Life Laboratory, Department of Women’s and Children’s Health, Karolinska Institutet, Solna, Sweden

**Keywords:** Apoptosis, Super-resolution microscopy

## Abstract

Activation of the apoptotic pathway is a major cause of progressive loss of function in chronic diseases such as neurodegenerative and diabetic kidney diseases. There is an unmet need for an anti-apoptotic drug that acts in the early stage of the apoptotic process. The multifunctional protein Na^+^,K^+^-ATPase has, in addition to its role as a transporter, a signaling function that is activated by its ligand, the cardiotonic steroid ouabain. Several lines of evidence suggest that sub-saturating concentrations of ouabain protect against apoptosis of renal epithelial cells, a common complication and major cause of death in diabetic patients. Here, we induced apoptosis in primary rat renal epithelial cells by exposing them to an elevated glucose concentration (20 mM) and visualized the early steps in the apoptotic process using super-resolution microscopy. Treatment with 10 nM ouabain interfered with the onset of the apoptotic process by inhibiting the activation of the BH3-only protein Bad and its translocation to mitochondria. This occurred before the pro-apoptotic protein Bax had been recruited to mitochondria. Two ouabain regulated and Akt activating Ca^2+^/calmodulin-dependent kinases were found to play an essential role in the ouabain anti-apoptotic effect. Our results set the stage for further exploration of ouabain as an anti-apoptotic drug in diabetic kidney disease as well as in other chronic diseases associated with excessive apoptosis.

## Introduction

The intrinsic apoptotic process of programmed cell death is a defense mechanism against cancer and plays a role in various aspects of tissue homeostasis and late embryonic development of the brain and kidneys [1]. However, it is inappropriately activated in certain diseases such as neurodegenerative diseases, where excessive apoptosis results in loss of neurons [2], and in type 2 diabetes, where the loss of pancreatic β-cells results in insulin deficiency [3]. The kidneys are also highly sensitive to inappropriate activation of the apoptotic pathway. Chronic kidney disease (CKD) is always progressive regardless of its origin [4], and ongoing apoptosis contributes to loss of renal epithelial cells and progressive deterioration of renal function [5, 6]. Diabetes is the most common cause of CKD. CKD affects 25% of all diabetic patients, and there is a great unmet need for therapy that halts its progression. Anti-apoptotic treatments with caspase inhibitors have been reported to suppress apoptosis and improve renal function in rodents [7]. However, despite great interest and numerous clinical trials, no caspase inhibitor has reached the drug market due to toxicity and poor pharmacokinetics [8]. Experience from failed trials indicates that future anti-apoptotic drug candidates must interfere with the apoptotic process upstream of caspase activation and the point of no return.

Our group has demonstrated that the cardiotonic steroid ouabain protects against apoptosis in rodent models of CKD [9] and Shiga toxin-exposed kidneys [10]. Ouabain is a highly specific ligand of the sodium pump Na^+^,K^+^-ATPase. High concentrations of ouabain inhibit the ion-pumping activity of Na^+^,K^+^-ATPase, while sub-saturating concentrations of ouabain enable Na^+^,K^+^-ATPase to function as a signal transducer [11–13]. Ouabain, a steroid found in the plant kingdom as well as in mammals [14], might be an attractive therapeutic candidate for conditions associated with inappropriate apoptosis, provided that its site-of-action within the apoptotic process is identified.

**Fig. 1:**
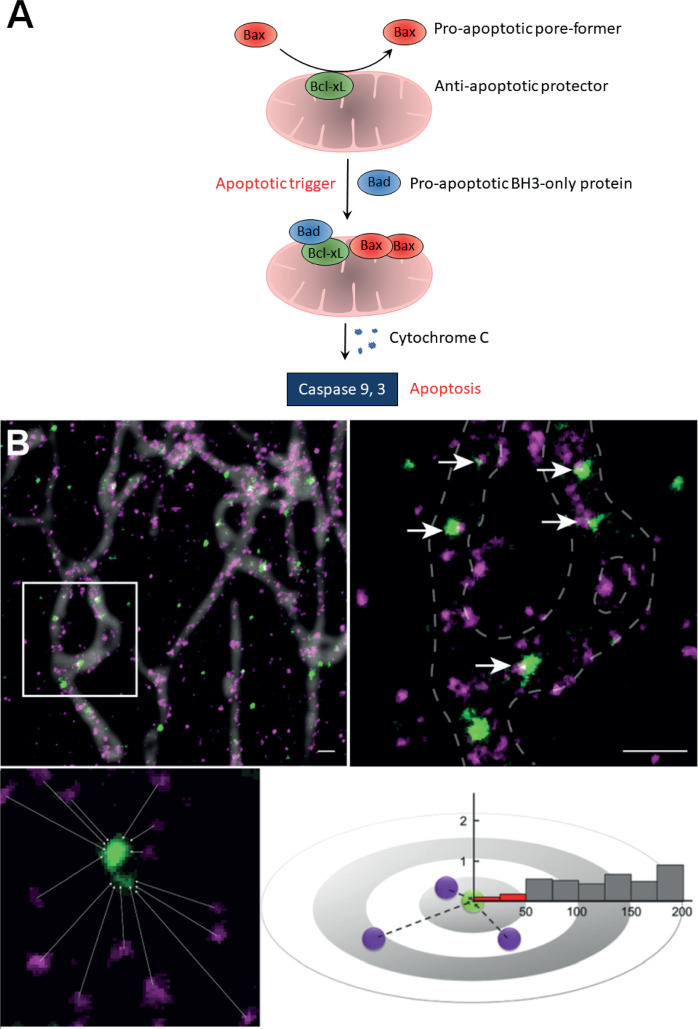
Schematic diagram of the apoptotic process and illustration of nearest neighbor analysis. **A** Schematic illustration of the apoptotic process governed by Bad, Bcl-xL, and Bax. All Bcl-2 family proteins contain at least one of the four Bcl-2 homology motifs (BH1–4), which are essential for their function. Pro-apoptotic BH3-only proteins (e.g., Bad, Bid, and Bik) only contain the BH3 domain, while pro-apoptotic pore-forming (Bax and Bak) and anti-apoptotic (Bcl-2, Bcl-xL, Bcl-w, Mcl-1, and Bfl-1/A1) proteins contain all four BH domains and are referred to as multi-domain proteins. In response to apoptotic stimuli, the BH3 domain of pro-apoptotic BH3-only proteins (Bad) binds to the BH3 domain-binding groove of anti-apoptotic Bcl-2 proteins (Bcl-xL). This disrupts binding between anti-apoptotic and pore-forming (Bax) proteins, causing activation of the pore-forming protein. Certain BH3-only proteins (Bid and Bim) can also induce apoptosis by directly binding to and activating pore-forming proteins on the outer mitochondrial membrane [15]. The point of no return in the apoptotic process occurs when apoptotic pore-forming proteins have accumulated on mitochondria and permeabilized the outer mitochondrial membrane, leading to the release of cytochrome c and activation of caspases present in the cytoplasm [19]. **B** Illustration of nearest neighbor analysis. The distances between two proteins, Bad (green) and Bcl-xL (magenta) in this example, are measured by super-resolution stimulated emission depletion (STED) microscopy and summarized in a histogram. The <50 nm distance fraction (red) between two species is indicative of molecular interaction. Scale bars = 500 nm.

The apoptotic process is governed by the Bcl-2 family of proteins, which comprises pro-apoptotic (either BH3-only or pore-forming proteins) and anti-apoptotic members that interact with one another [15]. Under control conditions, BH3-only proteins mainly reside in the cytoplasm, while anti-apoptotic proteins are mainly located on the mitochondrial membrane, and apoptotic pore-forming proteins are mainly located in the cytoplasm. The apoptotic proteins located on the mitochondrial membrane are maintained in an inactivated state by binding to anti-apoptotic proteins. The apoptotic process is initiated by the translocation of BH3-only proteins to the mitochondrial membrane, where they bind to the anti-apoptotic proteins. This disrupts the capacity of the anti-apoptotic proteins to bind to the pore-forming proteins. More pore-forming proteins are recruited to the mitochondria where they will penetrate the mitochondrial membrane, resulting in cytochrome C release, which marks the point of no return in the apoptotic process. Bad belongs to the subfamily of sensitizing BH3-only proteins [16] that initiate apoptosis by translocating to the mitochondrial membrane where they will form heterodimers with anti-apoptotic members of pa the Bcl-2 family. Bcl-xL is the only anti-apoptotic protein that can bind to and inactivate the apoptotic pore-forming protein Bax [16–18]. Bad/Bcl-xL interaction inhibits the capacity of Bcl-xL to maintain the pore-forming protein Bax in an inactive state and marks the beginning of the point of no return in the apoptotic process [19]. For a schematic illustration of the apoptotic process see Fig. 1A.

Edlich et al. [18] have in a groundbreaking publication visually described the time-dependent role of Bcl-2 proteins in the apoptotic process using fluorescence loss in photobleaching imaging of Bax and Bcl-xL. However, that study used transformed cells ectopically expressing fluorescently tagged proteins, and the results have not been confirmed in primary cells exposed to a clinically relevant challenge. Here we have used stimulated emission depletion (STED) microscopy and primary rat proximal tubule cells (PTCs) to examine whether ouabain can interfere with the apoptotic process before its point of no return. To provoke apoptosis, the cells have been exposed to high glucose concentrations. Hyperglycemia is the main cause of diabetic complications [20], and we have previously reported that a 2-fold increase of extracellular glucose concentration causes apoptosis in PTCs [21].

We identified the pro-apoptotic protein Bad as an early target for the anti-apoptotic effect of ouabain. The multifunctional protein kinase Akt, also known as protein kinase B, suppresses apoptotic death in several cell types via Bad inhibition [22, 23]. Akt is tightly controlled by its interacting molecules and by phosphorylation/dephosphorylation processes. Akt can be activated by ouabain [24, 25]. Although it is well recognized that the ubiquitous signaling molecule Akt exerts an anti-apoptotic effect by regulating Bad [22], there is relatively little information about the transduction of this survival pathway. Since ouabain signals with slow calcium oscillations in epithelial cells [11] and since calmodulin is particularly sensitive to an oscillatory calcium response [26], we have examined whether the ouabain survival signal may include Akt activation via calmodulin kinases.

## Materials and methods

### Animals and primary cell culture

Twenty-day-old male Sprague-Dawley rats were used to prepare primary cells. All animals were housed under controlled light and dark conditions (12 h:12 h), given a standard diet containing 20% protein by weight and provided tap water ad libitum. All experiments were performed according to the regulations of the Karolinska Institutet concerning the care and use of laboratory animals and were approved by the Stockholm North Ethical Evaluation Board for Animal Research.

Primary cultures of rat PTCs were prepared as previously described [9]. Animals were anesthetized by intraperitoneally injecting pentobarbital and the aorta was cut. The kidneys were removed and placed in 0.9% NaCl (Sigma-Aldrich) at 37 °C. The outer cortical layer (150 µm) was collected and incubated in basal solution, which comprised Hank’s Balanced Salt Solution containing 0.2% bovine serum albumin (BSA, Sigma-Aldrich), 10 mmol/l HEPES (Sigma-Aldrich), 10 µg/ml penicillin, 10 µg/ml streptomycin, and 0.035% collagenase I (Sigma-Aldrich), for 17 min while being carefully mixed with a sterile Pasteur pipette. Cells were washed twice with a basal solution containing 0.01% trypsin inhibitor and then with culture medium (pH 7.6), which comprised Dulbecco’s Modified Eagle Medium (DMEM) containing 20 mmol/l HEPES, 24 mmol/l NaHCO_3_ (Sigma-Aldrich), 10 µg/ml penicillin, and 10 µg/ml streptomycin. Cells were seeded in 12- or 24-well plates, with or without 12 or 18 mm glass coverslips at the bottom, and allowed to attach for 30 min, and then culture medium supplemented with 10% fetal bovine serum (FBS) was added. PTCs were cultured for 2–3 days at 37 °C under approximate humidity of 95–98% with 5% CO_2_. The culture medium was changed every day.

### In vitro model of hyperglycemia

PTCs were normally cultured in DMEM containing 5.6 mM d-glucose but were cultured in DMEM containing 20 mM d-glucose for high glucose treatment (HG treatment). The control medium was supplemented with an equimolar concentration of d-mannitol. All media contained 10% FBS. In some experiments, ouabain octahydrate (10 nM, Sigma-Aldrich) was added to the medium before use.

### Immunocytochemistry

Control and HG-treated PTCs were washed with phosphate-buffered saline (PBS), fixed with 4% paraformaldehyde for 10 min, and then permeabilized with 0.2% Triton X-100 prepared in PBS for 2 min. Immunostaining was performed overnight at 4 °C using primary antibodies against Bax (clone YTH-6A7, Trevigen, 2281-MC-100, 1:50 dilution), Bcl-xL (Cell Signaling, #2764, 1:200 dilution), and Bad (Santa Cruz Biotechnology, sc-8044, 1:100 dilution). All antibodies are well established and considered specific, verified by the high number of references to previous studies by other groups. Cells were then washed three times with PBS and incubated with the following secondary antibodies for 1 h at room temperature: Atto594 (goat anti-mouse IgG, Sigma-Aldrich, 76086, 1:500 dilution), Star635 (goat anti-mouse IgG, Abberior, 2-0012-007-2, 1:100 dilution), and GFP Tag Antibody, Alexa Fluor 488 conjugate (anti-rabbit, ThermoFisher Scientific, A-21311, 1:100 dilution).

### STED microscopy

Super-resolution STED imaging was performed using a Leica SP8 3X STED system equipped with lasers for the depletion of fluorophores emitting in the blue/green (592 nm, MPB Communications Inc.), orange (660 nm, Laser Quantum, USA), and red/far-red (775 nm, OneFive GmbH) ranges. A single depletion wavelength (660 nm) was applied for multi-color STED imaging of immunolabeled cells. Reshaping of the depletion beam was achieved with a vortex phase-plate in combination with an axial phase-ring to discriminate protein labeling on the mitochondrial structure. A chromatically optimized oil immersion objective (HC PL APO 100X/1.40 OIL STED White, Leica Microsystems) was used for imaging, and a tunable pulsed white light fiber-laser emitting at 470–670 nm was used for excitation. The selected excitation wavelength for the confocal mitochondrial reference was 488 nm. For the two super-resolved STED channels, wavelengths of 520 and 575 nm were used to excite Alexa Fluor 532 and Rhodamine Red X. The detected fluorescence signals were passed through a 0.8–0.09 Airy unit pinhole, a dichroic mirror optimized for the STED laser including notch filters placed in front of sensitive photodetectors (Leica Hybrid Detectors). The excitation pulse was also used to initiate gated detection of fluorescence and thereby further increase the focal depleted resolution by time gating. Multi-color frames (1024 × 1024 pixels) were acquired sequentially frame by frame at a scan speed of 600 lines per second with 8–16 line averages and a pixel size of 15 nm, with time gating initiated after 0.8–1.2 ns (closed after 6.5 ns). Precision calibrations were performed using coverslips with non-specifically attached secondary antibodies carrying a suitable fluorescent STED dye or with nano-sized fluorescent beads or DNA-origami structures. This indicated that resolved STED focal widths were about 40–50 nm.

### Nearest neighbor analysis

STED data were analyzed with custom-written scripts in MATLAB (Math Works Inc.). Through local water-shedding, individual clusters were located automatically, and the center was calculated and used for nearest neighbor analysis. For each Bcl-xL cluster located on mitochondria, the distance to the closest Bad or Bax cluster located on mitochondria was calculated. Briefly, a copy of each STED image was filtered through a Wiener filter and subsequently subjected to adaptive thresholding, where the threshold level was increased until separable regions could be distinguished. For each separate region, subsequent thresholding to further separate the regions was performed. These regions, if larger than 75 × 75 nm, were fed back into the loop until only small regions remained. These region outlines were then applied to the raw original data to extract the center of mass, which was used as an *xy* coordinate to describe the cluster and in nearest neighbor analysis.

The nearest neighbor analysis allows characterization of changes to protein–protein interactions in intact cells when used in combination with super-resolution imaging [27]. For each located particle, the distance to the closest particle of the second species is calculated and these distances are grouped in columns. The fraction of two species located within <50 nm of each other (distance fraction <50 nm, red) is considered to demonstrate a high likelihood of a molecular interaction. Distance fractions of 50–250 nm are shown in gray bars. Fig. 1B shows a schematic illustration of the nearest neighbor analysis.

### Assessment of apoptosis

Apoptosis was quantified by the TUNEL assay, which measures DNA fragmentation (late stage of apoptosis). Cells cultured on coverslips were fixed with ice-cold methanol, permeabilized with 33% acetic acid prepared in ethanol, and then labeled with an ApopTag Red In Situ Apoptosis Detection Kit (EMD Millipore) and DAPI. Images of TUNEL and DAPI staining were acquired by confocal or fluorescence microscopy. The total number of cells was calculated automatically using ImageJ software. The apoptotic index was calculated as the percentage of apoptotic cells.

### Reactive oxygen species (ROS) detection

ROS were measured with di(acetoxymethyl ester) 6-carboxy-2′,7′-dichlorodihydrofluorescein diacetate (DCFDA) (ThermoFisher Scientific). Intracellular ROS cause non-fluorescent DCFDA molecules to emit green fluorescence. Following glucose treatment, cells were incubated with 10 µM DCFDA for 30 min at 37 °C, rinsed twice with PBS, and then subjected to live cell imaging using a confocal microscope with fixed settings for all measurements. ROS were quantified as the mean DCFDA intensity in each image. At least eight individual areas were analyzed per coverslip.

### Mitochondrial membrane potential (ΔΨ_m_) measurement

Maintenance of ΔΨ_m_ was determined with JC-1 (Lifetime Technologies). JC-1 is a cationic carbocyanine dye that accumulates in mitochondria. At low concentrations, the dye is monomeric and emits green fluorescence (527 nm). As the concentration increases, the dye aggregates, which causes a fluorescence emission shift from green to red (590 nm). Following glucose treatment, cells were washed with Krebs-Ringer solution (pH 7.4), incubated in a culture medium containing 2.5 µg/ml JC-1 for 15 min at 37 °C, and then subjected to live cell imaging using a confocal microscope with fixed settings. Changes of ΔΨ_m_ were quantified by calculating the ratio of the red fluorescence intensity (polarized) to the green fluorescence intensity (depolarized) using MATLAB. Six separate areas were analyzed per coverslip. The values in all groups were normalized against that in the control.

### siRNA transfection

siRNA was transfected using Lipofectamine™ RNAiMAX Transfection Reagent (ThermoFisher Scientific, cat. no. 13778075) and Opti-MEM® I Reduced Serum Medium (ThermoFisher Scientific, cat. no. 31985062) according to the manufacturer’s instructions. In brief, cells were cultured in DMEM containing a low glucose concentration and 10% FBS. Opti-MEM-containing transfection reagents was added at a volume ratio of 1:10. CaMKK1 was silenced using Stealth siRNA (ThermoFisher Scientific, cat. no. RSS367727) and Bad was silenced using FlexiTube siRNA (Qiagen, Rn_Bad_2 FlexiTube). Stealth RNAi™ siRNA Negative Control, Med GC (ThermoFisher Scientific, cat. no. 12935300) was used as a control. Cells were transfected with 20 nM of the construct for 24 h. Thereafter, the transfection medium was replaced by a culture medium and cells were cultured for an additional 24 h prior to experiments.

### Quantitative real-time PCR

After 48 h of transfection, mRNA was extracted and purified using an mRNAeasy Mini Kit (Qiagen). The RNA concentration was determined using a Qubit™ RNA HS Assay Kit and a Qubit™ 3.0 fluorometer. Samples were subjected to one-step quantitative real-time RT-PCR using a Quant-X One-Step qRT-PCR SYBR Kit (Clontech) on a C1000 Touch™ Thermal Cycler (Bio-Rad). Each sample was analyzed in duplicate. Bad and CAMKK1 expression was analyzed using the ΔΔCt method described by Pfaffl [28], and GAPDH and RPL27 were used as housekeeping genes. The GAPDH and Bad primers were purchased from Qiagen (Rn_Gapd_1_SG and Rn_Bad_1_SG QuantiTect Primer Assays).

### Co-immunoprecipitation

The kidney cortex was obtained from 20-day-old male Sprague-Dawley rats as described in the “Animals and primary cell culture” sub-section. The slices were placed in a pre-labeling buffer containing 10 mM glucose and bubbled with 5% O_2_ and 95% CO_2_ for 10–20 min. Thereafter, the slices were treated with a pre-labeling buffer containing 10 or 30 mM glucose for 1 h. The cortex tissue was then lysed with RIPA buffer containing Pierce Protease and Phosphatase Inhibitor Mini Tablets (ThermoFisher, cat. no. 88669). Samples were then homogenized and centrifuged to remove debris.

For immunoprecipitation, the anti-Bad 11E3 antibody (Cell Signaling, #9268) or normal rabbit IgG was bound to 50 μl of Dynabeads (ThermoFisher Scientific, 10004D). The bead and antibody mixture was incubated with rotation for 1 h at room temperature. The beads were then washed and incubated with tissue lysates (0.8 mg of protein per sample) overnight at 4 °C. The beads were washed again, and proteins were extracted with 50 µl of SDS buffer (62.5 mM Tris-HCl, pH 6.8, 2% SDS, and 10% glycerol). The samples were heated at 70 °C for 15 min and then subjected to western blot analysis. The optical density of Akt in treated groups was normalized to that of untreated controls.

### Immunoblotting

For western blotting, cells were cultured and treated in low glucose DMEM supplemented with antibiotics, 10% FBS, and growth factor (PDGF-CC, 10 ng/ml) Samples were loaded onto 7.5% Mini-PROTEAN® TGX™ Precast Protein Gels (Bio-Rad, #4561023). Electrophoresis and transfer were performed using a Bio-Rad electrophoresis and transfer system. The membrane was blocked with Tris-buffered saline (TBS) containing 5% BSA or 0.1% casein in TBS-T and then incubated with a primary antibody targeting Akt1 (C73H10, cat. no. #2938, Cell Signaling, 1:1000 dilution), CaMKKα (F-2, cat. no. sc-17827, Santa Cruz Biotechnology, 1:1000 dilution), Bad (Y208, cat. no. ab32445, Abcam, 1:500 dilution), Phospho-Bad (Ser136) (D25H8, cat. no. 4366S, Cell Signaling, 1:500 dilution), Anti-AKT phospho T308 (cat. no. ab38449, Abcam, 1:500 dilution), Phospho-Akt Ser473 (cat. no. 9271, Cell signaling, 1:500 dilution), GAPDH (6C5, cat. no. sc-32233, Santa Cruz Biotechnology, 1:2000 dilution) or α-tubulin (cat. no. T6074, MilliporeSigma, 1:2000 dilution) overnight at 4 °C followed by a secondary antibody (TrueBlot® Anti-Rabbit Ig, cat. no. 18-8816-33, Rockland Immunochemicals or Mouse IgG HRP Linked Whole Ab cat. no. GENXA931, GE Healthcare at 1:2000 dilution) for 2 h at room temperature. Both antibodies were diluted in TBS-T (0.1% Tween 20 prepared in TBS) containing 5% BSA. Immunoreactive bands were visualized by SuperSignal West Femto chemiluminescence reagent (ThermoFisher Scientific) and images were acquired using an Odyssey Imaging System (LI-COR). When needed, membranes were stripped using Restore™ Western Blot Stripping Buffer (ThermoFisher) for 15 min, blocked, and re-probed.

### Quantification and statistical analysis

STED data were analyzed with custom-written scripts in MATLAB® (Math Works Inc.). Through local water-shedding, individual clusters were located automatically, and the center was calculated and used for nearest neighbor analysis. For each Bcl-xL cluster located on mitochondria, the distance to the closest Bad or Bax cluster located on mitochondria was calculated (Fig. 1A).

Other summarized data are presented as mean ± SEM. Statistical significance was calculated using the Student’s two-tailed t-test for paired comparisons and an ANOVA for unpaired comparisons (Figs. 4C, 6F, G, 7C and Supplemental Fig. 1A–C) with Tukey’s multiple comparison test for post hoc analysis. P values < 0.05 were considered statistically significant.

## Results

### Exposure to a high glucose concentration triggers apoptosis of renal epithelial cells in a time- and dose-dependent manner

This study was performed on rat primary PTCs, which mainly take up glucose via the sodium-dependent glucose transporter SGLT2 [29]. Cells were grown in a solution containing 5.6 mM glucose. Exposure to 10, 20, or 30 mM glucose for 6 h dose-dependently increased the apoptotic index, as determined by TUNEL (Supplemental Fig. 1A). In subsequent experiments, cells were challenged with 20 mM glucose (HG) (Supplemental Fig. 1B). Signs of mitochondrial stress such as moderately increased levels of reactive oxygen species and depolarization of the mitochondrial membrane were observed already after 2 h of HG exposure (Supplemental Fig. 1C, D). Remodeling and fragmentation of the mitochondrial network may be observed during apoptosis. Supplemental Fig. 1E shows mitochondria visualized using mitochondrial-targeted GFP in control and HG-exposed cells. Control cells had a reticulated and elongated mitochondrial network. This network remained intact after HG exposure for 2 h but tended to have shorter rods and more frequent rounded shapes with a small radius of curvature after HG exposure for 4 h (Supplemental Fig. 1E).

### High glucose treatment shifts the balance between cytosolic and mitochondrial Bax

We first studied the effects of HG treatment on the locations of Bax and Bcl-xL and their interaction on mitochondria to confirm their involvement in the apoptotic effects of HG exposure and to validate the visualization of the process using STED microscopy. Interactions/heterodimerization between key members of the Bcl-2 family were semi-quantitatively recorded using the nearest neighbor algorithm [27]. Figure 1A shows a schematic illustration of the investigated apoptotic process.

**Fig. 2:**
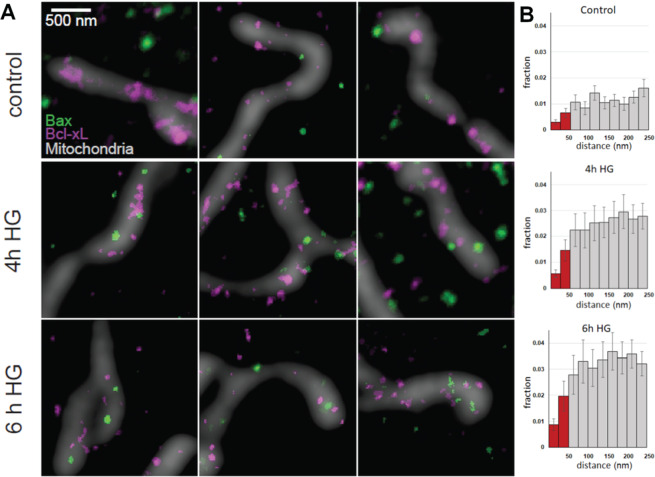
Stimulated emission depletion (STED) micrographs and nearest neighbor analysis of mitochondria, Bcl-xL, and Bax in high glucose (HG)-treated proximal tubular cells (PTCs). **A** Representative images showing the distributions of Bax (green) and Bcl-xL (magenta) together with mitochondria (gray). The top row shows untreated (5.6 mM glucose) cells. The second and third rows show cells treated with HG for 4 and 6 h, respectively. **B** Nearest neighbor analysis of interactions based on the accumulated statistics for distances between Bcl-xL and Bax measured by super-resolution STED microscopy. Bars show the fractions of proteins with a separating distance indicated at *x* axis. The <50 nm distance fraction (red) is considered indicative of a molecular interaction. N = number of micrographs from different cells obtained from 3 or more cell culture preparations; 31 in control, 36 in 4 h HG, and 28 in 6 h HG.

In control cells, Bax was mainly cytosolic, but a small fraction was located at mitochondria (Fig. 2A), consistent with a previous report [18]. Cells exposed to HG displayed an increase in mitochondrial Bax abundance and a 3-fold increase (*p* = 0.003) in the fraction of Bax and Bcl-xL localized within <50 nm of each other (<50 nm distance fraction) after 6 h (Fig. 2A, B), which would be symptomatic of apoptosis.

### HG treatment triggers translocation of Bad to mitochondria and its interaction with Bcl-xL

**Fig. 3:**
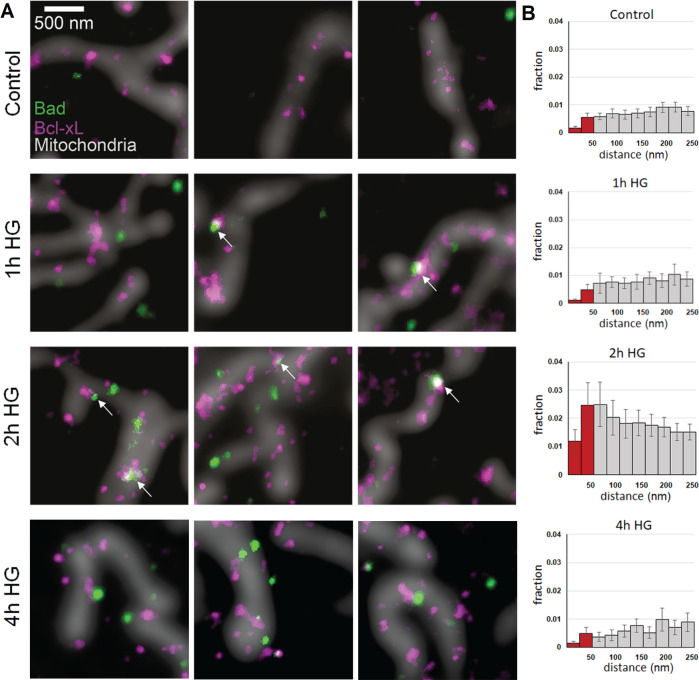
Bad translocates toward mitochondrial Bcl-xL in proximal tubular cells (PTCs) upon high glucose (HG) treatment. **A** Representative stimulated emission depletion (STED) images showing the distributions of Bad (green) and Bcl-xL (magenta) in PTCs treated with HG or 5.6 mM glucose (Control) together with confocal images of mitochondria (gray). Arrows indicate colocalization of proteins. **B** Nearest neighbor analysis of the data presented in **A**. *N* = number of micrographs from different cells obtained from 3 or more cell culture preparations; 56 in control, 62 in 2 h HG, and 26 in 4 h HG.

To study the mitochondrial translocation of Bad and its interaction with Bcl-xL, STED microscopy was performed on PTCs immune-stained for Bad and Bcl-xL in the presence or absence of HG. Micrographs acquired after HG treatment for 1, 2, and 4 h showed that Bcl-xL localized to mitochondria at all time points (Fig. 3A). Bad was not located in close proximity to Bcl-xL or mitochondria under control conditions, but the mitochondrial level of Bad increased dramatically after HG treatment for 2 h (Fig. 3B). This was reflected by a 5-fold increase in the <50 nm distance fraction (*p* = 0.004). The increase in the association between Bad and Bcl-xL subsided after HG treatment for 4 h.

**Fig. 4:**
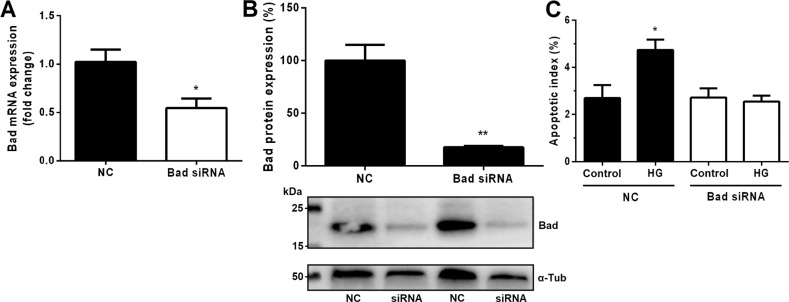
Bad is critical for glucose-induced apoptosis. **A** Quantitative real-time PCR demonstrates that transfection of Bad-targeting siRNA reduces mRNA expression of Bad by about 50% compared with cells transfected with scrambled negative control (NC) construct. *N* = 3. **B** Western blot of transfected cells showing downregulation of Bad protein by 82%. *N* = 3. **C** TUNEL shows that the percentage of apoptotic proximal tubular cells (PTCs) following high glucose (HG) treatment for 8 h significantly increases among cells transfected with the NC construct but not among those transfected with Bad-targeting siRNA. *N* = 6–8. *p < 0.05 and **p < 0.01 vs. control.

Since members of the Bcl-2 family often show a high degree of functional redundancy, we next investigated whether Bad is required for apoptosis induced by exposure to high glucose concentration by silencing its expression in PTCs using siRNA (Fig. 4A, B). Cells were treated with HG for 8 h, and the degree of apoptosis was determined by the TUNEL assay (Fig. 4C). HG treatment significantly increased the apoptotic index in control cells transfected with a scrambled construct (NC). By contrast, cells transfected with Bad-targeting siRNA did not respond to HG treatment, demonstrating that Bad is critical for glucose-mediated apoptosis of PTCs (Fig. 4C). Micrographs from the TUNEL assay can be seen in Supplemental Fig. 2A.

### Ouabain prevents glucose-induced translocation of Bad and Bax to mitochondria

**Fig. 5:**
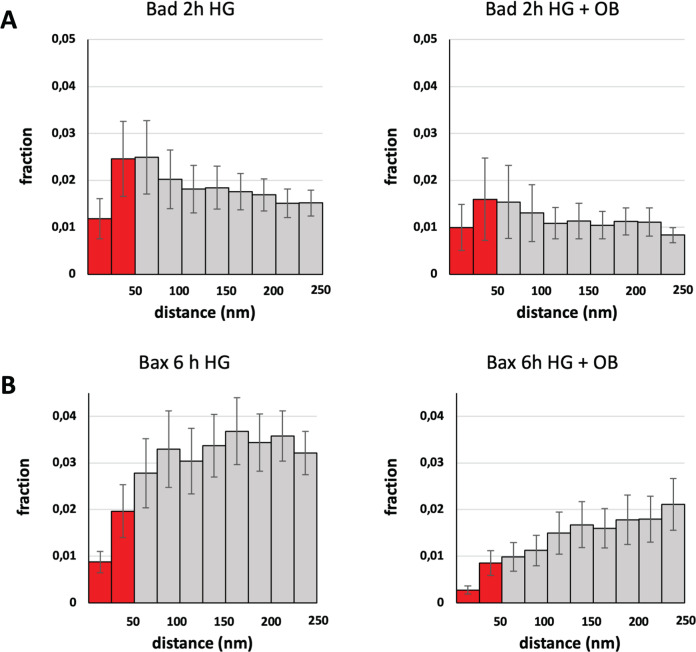
Ouabain prevents glucose-induced translocation of Bad and Bax to mitochondria. **A** Nearest neighbor analysis of Bad and Bcl-xL in cells exposed to high glucose (HG) for 2 h with or without ouabain (OB). (For control see Fig. 3B). **B** Nearest neighbor analysis of Bax and Bcl-xL in cells exposed to high glucose for 6 h with or without ouabain. (For control see Fig. 2B). The analysis confirms that ouabain effectively prevents translocation of Bax to mitochondria.

Next, we studied the effects of a sub-saturating concentration of ouabain on the apoptotic process initiated by HG. We first assessed the effects of 10 nM ouabain on the glucose-induced interaction between Bad and Bcl-xL using STED microscopy together with nearest neighbor analysis. Ouabain reduced the interaction between Bad and Bcl-xL following HG treatment for 2 h; however, this effect was not significant (Fig. 5A). Ouabain dramatically attenuated the glucose-induced translocation of Bax to mitochondria and Bcl-xL after 6 h, which was reflected by a 3-fold reduction in the <50 nm distance fraction (*p* = 0.006) (Fig. 5B). Together, our results demonstrate that ouabain effectively modulates these Bcl-2 family proteins to inhibit apoptosis

### Ouabain elicits its anti-apoptotic effects via Akt and calmodulin-dependent protein kinases

**Fig. 6:**
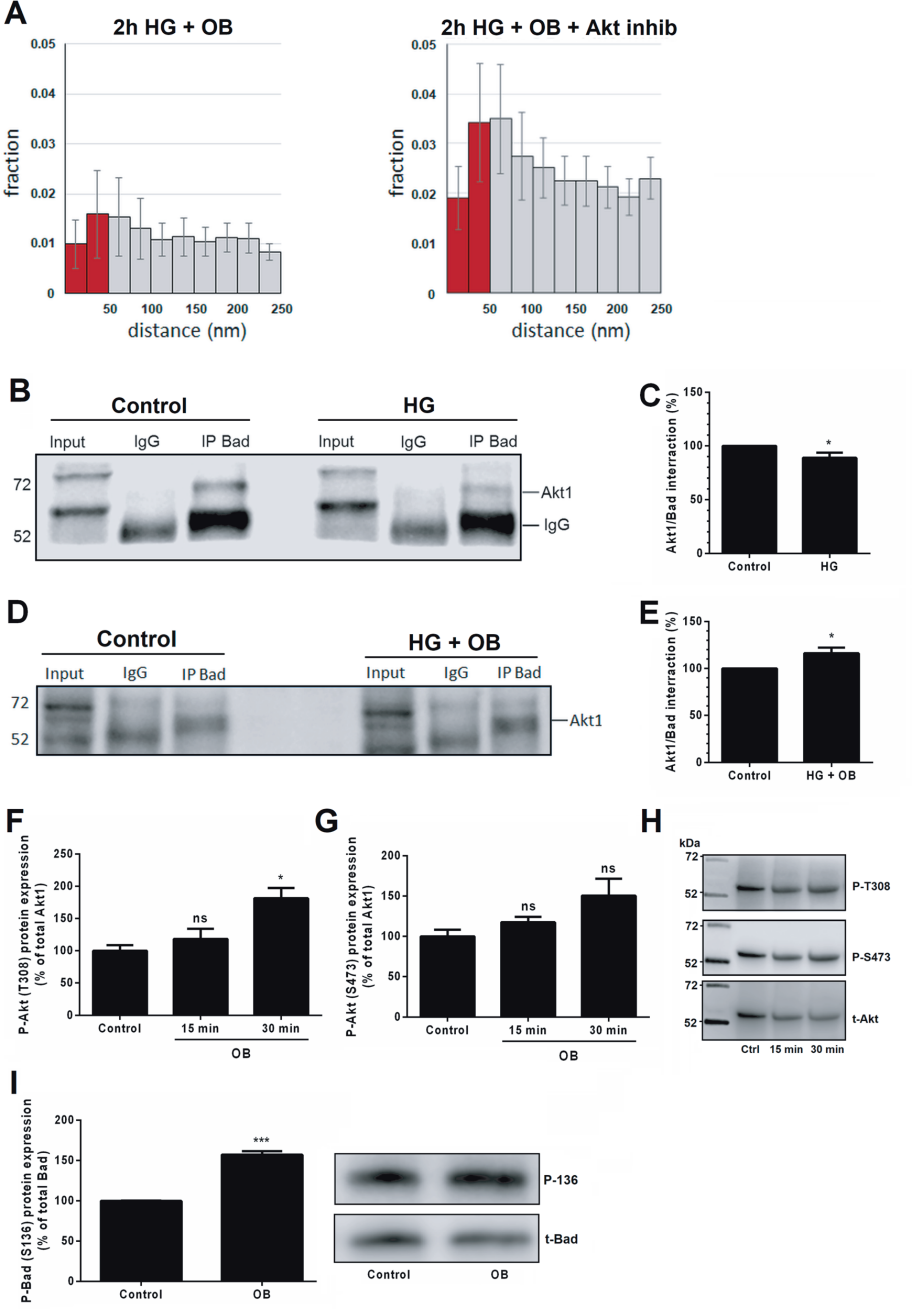
Akt is required for the anti-apoptotic effect of ouabain. **A** Nearest neighbor analysis of Bad and Bcl-xL using stimulated emission depletion (STED) images acquired after treatment with HG and 10 nM ouabain together with an Akt inhibitor. The right panel shows that inhibition of Akt counteracts the disruptive effect of ouabain on translocation of Bad to mitochondria upon HG treatment. *N* = 30 different cell recordings. (For control see Fig. 3B). **B** Immunoblot of an immunoprecipitate obtained with an anti-Bad antibody from rat kidney cortex treated with high glucose (HG) or low glucose. Akt1 co-immunoprecipitates with Bad in both groups. “Input” refers to tissue lysate prior to immunoprecipitation. “IgG” refers to the immunoprecipitate obtained with control IgG. **C** The relative amount of Akt1 protein was determined by densitometric analysis of immunoblots. The amount of Akt1 that immunoprecipitates with Bad are reduced upon HG treatment, indicating that the interaction between these two proteins is reduced and thus inhibition of pro-apoptotic Bad is perturbed. *N* = 6 different tissue lysates. **D** Immunoblot of an immunoprecipitate obtained with an anti-Bad antibody after treatment with or without HG and ouabain. **E** Densitometric analysis of Akt1 immunoreactivity reveals that the interaction between Akt1 and Bad is increased by ouabain, indicating that the inactivation of Bad by Akt is enhanced. *N* = 5 different tissue lysates. **F** Western blot of phospho-Akt T308 shows increased phosphorylation by 81% after 30 min of ouabain treatment. *N* = 3. **G** Western blot of phospho-Akt S473 shows a similar trend as T308 although not significant. *N* = 3. **H** Immunoreactive bands of **F** and **G** after stripping and re-probing. **I** Western blot of phospho-Bad S136 after 15 min of ouabain treatment shows enhanced phosphorylation by 57%. *N* = 3. *p < 0.05 and ***p < 0.001 vs. controls.

To study the role of Akt in the anti-apoptotic effect of ouabain, we treated PTCs with HG and 10 nM ouabain in the presence or absence of the Akt inhibitor MK-2206 (5 µM) and imaged these cells by STED microscopy. Akt inhibition reduced the capacity of ouabain to attenuate colocalization of Bad and Bcl-xL in HG-treated cells (Fig. 6A). To confirm the involvement of Akt in the HG-dependent apoptotic process we immunoprecipitated Bad from rat kidney cortex treated with 10 or 30 mM glucose and immunoblotted for Akt1, the predominant Akt isoform involved in Bad phosphorylation [30]. Akt1 immunoprecipitated with Bad, and pre-treatment of kidney cortical tissue with 30 mM glucose reduced the Akt-Bad interaction by 11% (*p* = 0.044, Fig. 6B, C). Pre-treatment of kidney cortex with 10 or 30 mM glucose in the presence of ouabain enhanced the Akt-Bad interaction by 18% in samples treated with 30 mM glucose (*p* = 0.026, Fig. 6D, E). Akt is activated by coordinated phosphorylation of S473 and T308 [30]. We found that ouabain treatment resulted in significant phosphorylation of T308 with a similar effect on S473 although not significant (Fig. 6F–H). Additionally, we investigated the phosphorylation state of Bad S136 following ouabain treatment and noted an increase of phosphorylated protein by ~60% (*p* = 0.0002, Fig. 5I). Taken together these results indicate that the anti-apoptotic effect of ouabain involves Akt activation.

**Fig. 7:**
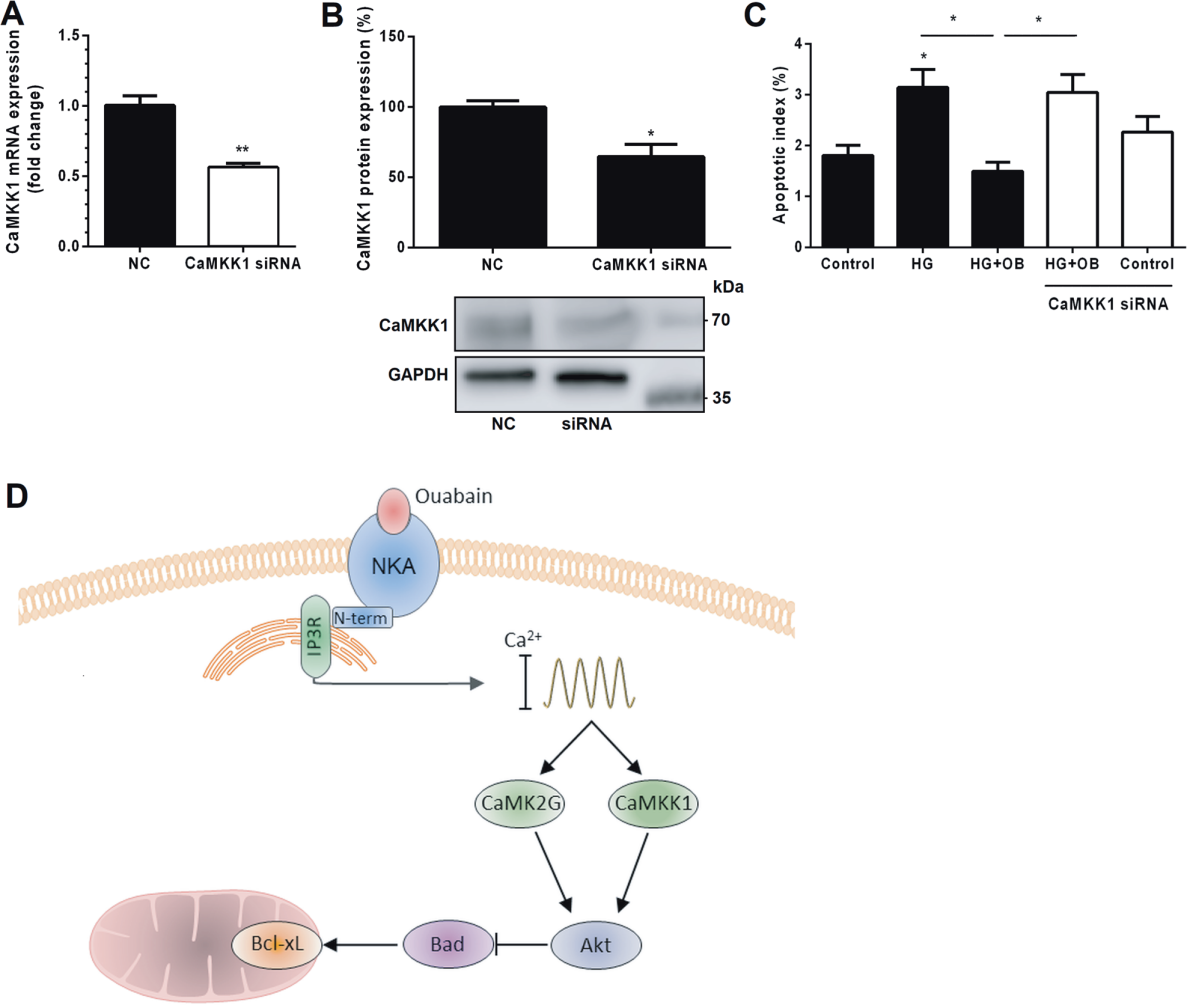
Ouabain elicits anti-apoptotic effects through CaMKK1 signaling. **A** CaMKK1 was silenced in rat proximal tubular cells (PTCs). RT-qPCR shows that mRNA expression of CaMKK1 is reduced by about 40% after siRNA treatment. *N* = 3. **B** Western blot of transfected cells showing downregulation of CaMKK1 by 35%. *N* = 3. **C** TUNEL assay of PTCs challenged with high glucose (HG) with or without ouabain and CaMKK1-targeting siRNA. Ouabain abolishes HG-induced apoptosis in control cells but not in those transfected with CaMKK1-targeting siRNA. *N* = 3. *p < 0.05 and **p < 0.01 vs. controls. **D** Schematic presentation of the proposed signaling cascade by which ouabain protects against apoptosis. Binding of ouabain to Na^+^,K^+^-ATPase results in concerted activation of the inositol 1,4,5-trisphosphate receptor (IP3R) and store-operated calcium channels regulated by STIM [34]. Calcium oscillations are subsequently generated by the endoplasmic reticulum. This activates CaM/Ca^2+^, which in turn binds to and activates CaMKK1 and/or CaMK2G. CaMKK1 and CaMK2G activate Akt via phosphorylation (T308 [23] and S473 [32], respectively). Akt phosphorylates Bad at S136, resulting in sequestration of Bad by 14-3-3 protein and inactivation of its pro-apoptotic properties.

Ca^2+^/CaM-dependent kinases (CaMKs) can activate Akt, either directly or via facilitating activation of Erk by calcium. Both pathways result in phosphorylation of Bad and subsequent repression of apoptosis [23, 31–33]. In a recent phosphoproteomic study, we demonstrated that ouabain regulates CaMK2G and CaMKK1 and showed that downregulation of CaMK2G abrogates the anti-apoptotic effect of ouabain in cells challenged with serum deprivation or HG exposure [34]. Here, we have investigated whether CaMKK1 is also required for the anti-apoptotic effect of ouabain. Rat proximal tubular cells were depleted of CaMKK1 using siRNA, which reduced its mRNA and protein levels by 40% and 35%, respectively (Fig. 7A, B). The cells were treated with or without HG and ouabain for 6 h and the degree of apoptosis was estimated using TUNEL assay (for micrographs see Supplemental Fig. 2B). HG treatment significantly increased the apoptotic index in both control and siRNA-treated cells. Ouabain (10 nM) rescued from the apoptotic effect of HG in control cells, but not in cells transfected with CaMKK1-targeting siRNA (Fig. 7C).

## Discussion

We have demonstrated the applicability of ouabain as an anti-apoptotic drug by studying its effects on the initial phase of glucose-triggered apoptosis using super-resolution imaging. Our study is the first reproduction of the apoptotic process as it occurs in primary mammalian cells. Our methodological approach made it possible to visualize the movement of the endogenously expressed pro-apoptotic protein Bad and the pro-apoptotic protein Bax from the cytoplasm to the mitochondria in the early phase of the apoptotic process. Increased extracellular glucose concentration triggered translocation of Bad to the mitochondrial membrane and association with Bcl-xL within 2 h after HG exposure. Recruitment of the pore-forming apoptotic protein Bax to the mitochondria occurred 4 h after HG exposure. Ouabain abolished Bax transfer to the mitochondria, which is considered as the point of no return in the apoptotic process. New steps in the signaling pathway by which ouabain exerts this anti-apoptotic effect have been identified.

Colocalization analysis of proteins is often used to identify possible protein interactions and signaling cascades. However, due to the limited spatial resolution of classical fluorescence microscopy, the fusion of colors indicative of colocalization occurs already at distances of 200–300 nm [27, 35]. The development of super‐resolution fluorescence imaging has made it possible to study the localization of proteins at a much higher resolution and prompted the development of new methods for the analysis of colocalization. Nearest neighbor analysis [27] allowed us to quantify interprotein relationships with much greater precision than traditional techniques and with a lower risk of artifacts than spatial correlation analysis. Edlich et al. visually described the time-dependent role of the Bcl-2 proteins Bax and Bcl-xL in the apoptotic process. They visualized the localization of Bcl-2 family proteins using fluorescence loss in photobleaching confocal microscopy [18]. A weakness of this methodological approach is that it requires the transfection of cells and can only visualize ectopically expressed fluorescently tagged proteins. By contrast, our approach allows the visualization of native proteins in primary cells exposed to a clinically relevant challenge.

We used Bad as an exponent of BH3-only proteins in this study of glucose-driven apoptosis for several reasons. The binding of Bad to Bcl-xL and inhibition of the anti-apoptotic effects of Bcl-xL is well documented [36, 37]. In addition, Bad has interesting properties related to glucose metabolism. Bad phosphorylated on its BH3 domain stimulates glucose-driven mitochondrial respiration and plays a role in glucose-stimulated insulin secretion by pancreatic β-cells [38, 39].

The multifunctional protein kinase Akt has been known for more than 20 years to exert an anti-apoptotic effect by its capacity to regulate Bad [22, 30], but the sequence of regulatory steps in this survival signal has not been fully elucidated. It has been reported that Akt-regulation of survival requires activation of CaMKK1 kinase [33] and CaMK2G [40], but the mechanism of activation of these two kinases and the question of whether they are interchangeable has not been addressed. Ouabain activates a slow oscillatory calcium signal (Supplemental Fig. 3) that provides a high level of specificity of the calcium signal [11]. Calmodulin is known to be particularly sensitive to calcium oscillatory signals. Recently we demonstrated that both CaMKK1 and CaMK2G are transiently phosphorylated and dephosphorylated by ouabain. Taken together our results are compatible with the hypothesis that ouabain triggers the Akt-Bad survival signal by activation of both CaMK2G and CaMKK1 (Fig. 7D).

Ouabain binds specifically to the catalytic Na^+^,K^+^-ATPase subunit [41], at high concentrations it inhibits the transporting activity of Na^+^,K^+^-ATPase. In clinical medicine low concentrations of the cardiotonic steroid digitalis have been used to treat heart failure and atrial fibrillation [42]. Sub-saturating concentrations of ouabain elicit an anti-apoptotic effect in vascular smooth muscle cells [43], endothelial cells [44], neurons [45, 46], and kidney cells [9, 47]. Ouabain protects kidney cells in embryos exposed to starvation [48] and in adult mice with hemolytic anemia caused by Shiga toxin [10]. Interestingly, ouabain appears to show selective toxicity toward cancer cells over primary cells [49]. This stresses the importance of using primary cells over cancer cell lines to study the physiological effects of ouabain anywhere but in tumors.

High glucose causes apoptosis in kidney epithelial cells and in pancreatic beta cells [3]. Kidney cell apoptosis is a major cause of diabetic nephropathy, which today is the most common cause of renal failure. Diabetic complications are examples of disorders where there is an unmet need for anti-apoptotic therapy. Understanding each step in the apoptotic process that leads to loss of functional cells is critical for the development of anti-apoptotic therapy. Ouabain, which exists as an endogenous compound and inhibits the apoptotic process before the recruitment of the pore-forming apoptotic proteins to the mitochondria, should be considered as a promising anti-apoptotic drug.

## Supplementary information

Supplemental figures 1,2,3
