# Supplementary material for: Super-resolution microscopy reveals that Na+/K+-ATPase signaling protects against glucose-induced apoptosis by deactivating Bad
Source: Cell Death Dis. 2021 Jul 27;12(8):739. doi: 10.1038/s41419-021-04025-8 (PMC8316575; doi:10.1038/s41419-021-04025-8)
Supplement: Supplementary file 1 — Supplemental figures 1,2,3 [file 41419_2021_4025_MOESM1_ESM.pdf]

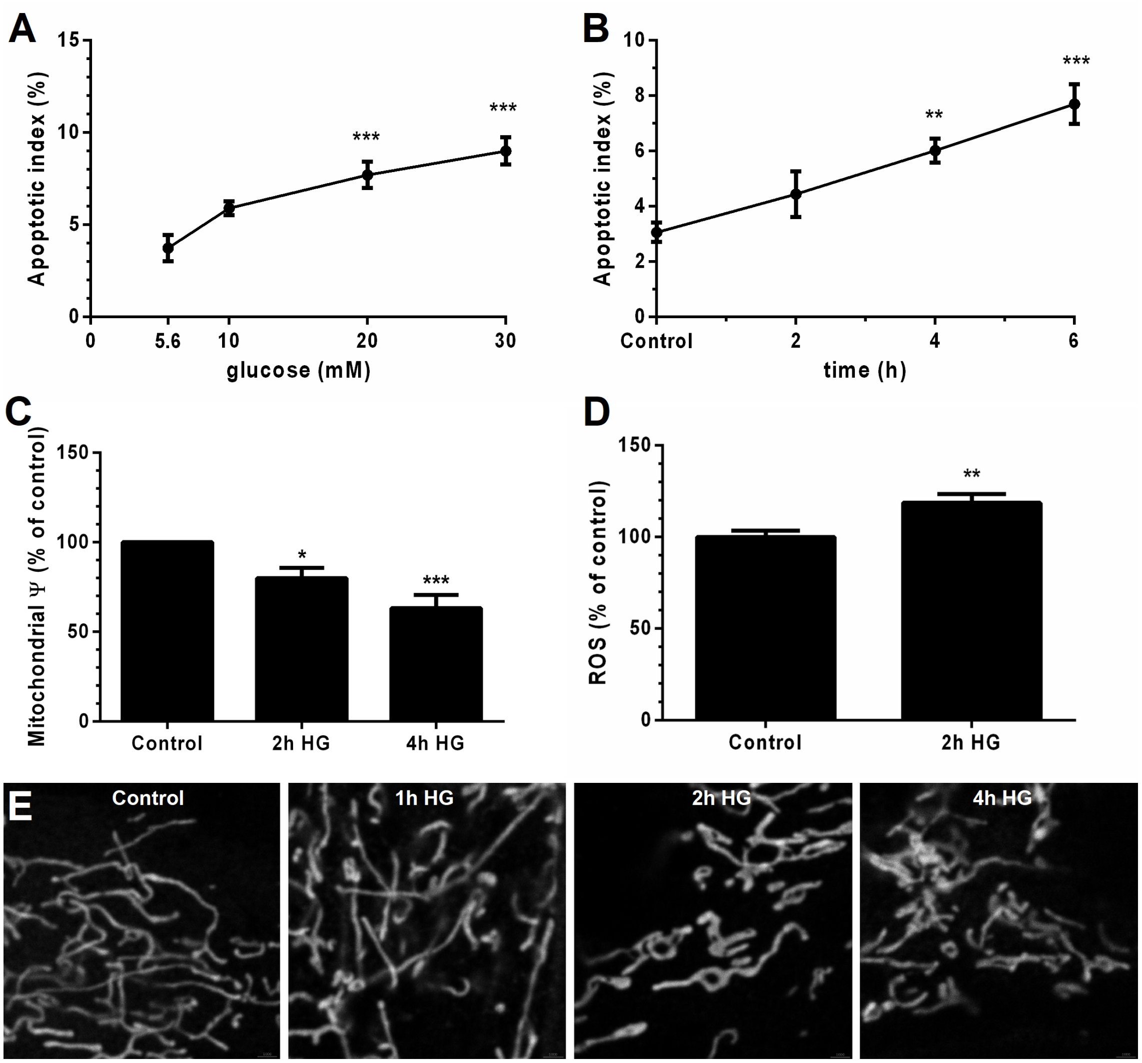

Supplemental Figure 1. High glucose (HG) treatment triggers apoptosis and alters mitochondrial morphology and function. (**A, B**) HG treatment induces apoptosis of proximal tubular cells (PTCs) in a dose- and time-dependent manner, as determined by the TUNEL assay. The dose response was determined at 6 h, and the time response was determined with 20 mM glucose. (**C**) High-resolution confocal images of mitochondria in HG-treated cells indicate shape changes. HG-treated mitochondria appear to undergo fission and form aggregates. (**D**) Mitochondrial membrane potential is significantly reduced by HG after 2 h and is decreased by about 40% after 4 h. N = 6. (**E**) Semi-quantification of reactive oxygen species (ROS) reveals that ROS production is increased by about 20% in cells treated with HG for 2 h. N = 40. * $p < 0.05$, ** $p < 0.01$, and *** $p < 0.001$ *vs*. controls.

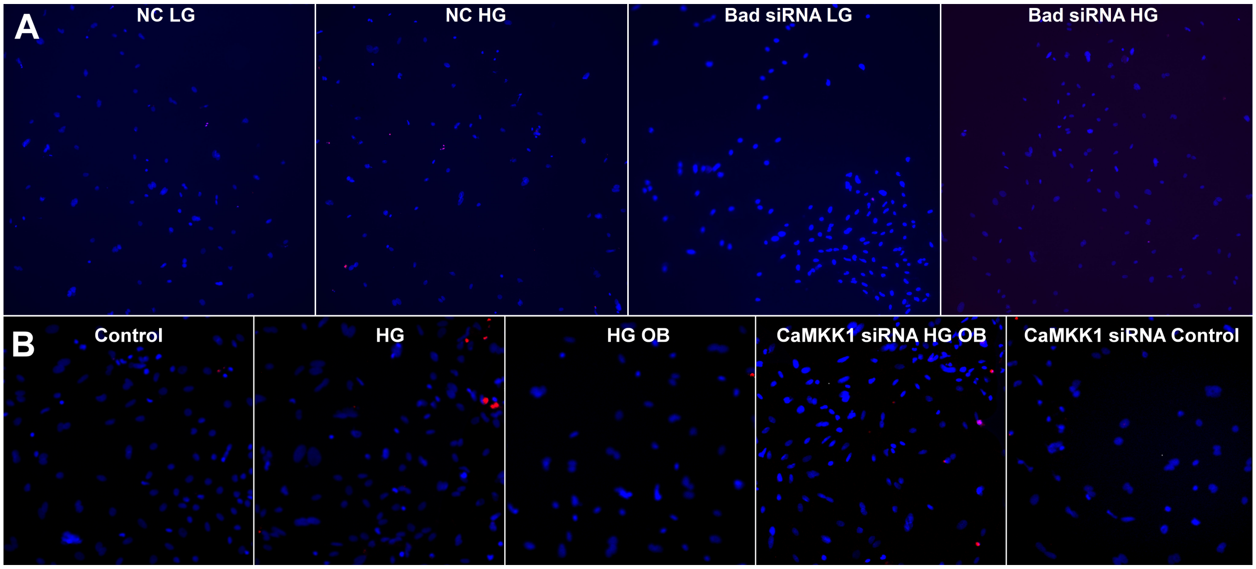

Supplemental Figure 2. Micrographs acquired in the TUNEL assay after treatment with ouabain and HG in cells transfected with or without Bad siRNA (A) or CaMKK1 siRNA (B). TUNEL staining, indicating DNA fragmentation and apoptosis is stained red. Nuclei staining with DAPI is shown in blue.

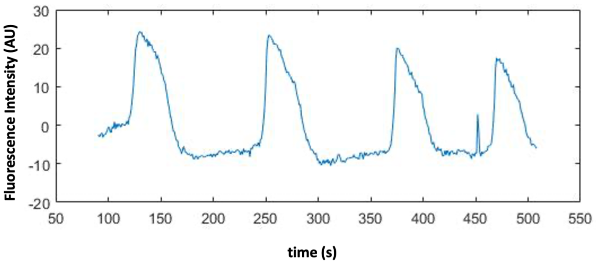

Supplemental Figure 3. Ouabain induces calcium-oscillations in rat PTCs. Measurement of fluorescence intensity (arbitrary units) emitted by the calcium-sensitive dye Fura Red in a single cell treated with 100 nM of ouabain over time (s).
